# Adjunctive brexpiprazole in patients with major depressive disorder and anxiety symptoms: an exploratory study

**DOI:** 10.1002/brb3.520

**Published:** 2016-07-24

**Authors:** Lori L. Davis, Ai Ota, Pamela Perry, Kana Tsuneyoshi, Emmanuelle Weiller, Ross A. Baker

**Affiliations:** ^1^ Veterans Affairs Medical CenterTuscaloosaALUSA; ^2^ University of Alabama School of MedicineBirminghamALUSA; ^3^ Otsuka Pharmaceutical Co., Ltd.TokyoJapan; ^4^ Otsuka Pharmaceutical Development & Commercialization, Inc.PrincetonNJUSA; ^5^ H. Lundbeck A/SValbyDenmark

**Keywords:** antidepressant treatments, anxiety, clinical trials, depression, mood disorders

## Abstract

**Background:**

Major depressive disorder (MDD) with concurrent anxiety symptoms may signal a difficult‐to‐treat patient. Brexpiprazole is a serotonin–dopamine activity modulator: a partial agonist at 5‐HT_1A_ and dopamine D_2_ receptors at similar potency, and an antagonist at 5‐HT_2A_ and noradrenaline alpha_1B/2C_ receptors. The objective of this Phase IIIb study was to explore effectiveness, safety, and tolerability of brexpiprazole adjunctive to antidepressant (ADT) monotherapy in patients with MDD and anxiety symptoms (NCT02013531).

**Methods:**

Patients with MDD, Hamilton Anxiety Rating Scale (HAM‐A) total score ≥ 20, and inadequate response to current ADT received open‐label brexpiprazole 1–3 mg day^−1^ (target dose 2 mg day^−1^) + ADT for 6 weeks. Efficacy endpoints included change from baseline at Week 6 in Montgomery–Åsberg Depression Rating Scale (MADRS) total score, HAM‐A total score, and Sheehan Disability Scale (SDS). Safety and tolerability assessments included adverse events (AEs).

**Results:**

Of 37 participants enrolled, 32 (86.5%) completed the study. Baseline mean (*SD*) MADRS total score was 30.1 (5.1); mean HAM‐A total score was 26.9 (5.0). Improvements from baseline were observed at Week 6 for least squares mean change in MADRS total score (−19.6, *p *< .0001 vs. baseline), HAM‐A total score (−17.8, *p* < .0001) and mean (*SD*) SDS mean score [−3.6 (2.6)]. Brexpiprazole was well tolerated. The most frequent treatment‐emergent AEs were increased appetite (13.5%) and diarrhea, dry mouth, and dizziness (all 10.8%).

**Conclusions:**

These open‐label results support the anxiolytic effects of adjunctive brexpiprazole in the treatment of patients with MDD.

## 1 Introduction

Anxiety symptoms are common in patients with major depressive disorder (MDD) (Fava et al., 2000; Zimmerman, McDermut, & Mattia, 2000). Many studies have shown that anxiety symptoms in patients with MDD are associated with more severe depression, poorer course, greater impairment in functioning, and worse health‐related quality of life (Fichter et al., 2010; Rhebergen et al., 2011; Wiethoff et al., 2010; Zimmerman et al., 2014), suggesting that anxiety symptoms in MDD are an indicator for more difficult‐to‐treat patients. Indeed, the clinical significance of the presence of anxiety symptoms was highlighted in the most recent *Diagnostic and Statistical Manual of Mental Disorders*, Fifth Edition (DSM‐5), which included the addition of criteria for a “with Anxious Distress” specifier for MDD (American Psychiatric Association, 2013).

Evidence‐based guidance on treatment strategies for patients with MDD and concurrent anxiety symptoms is scarce. Several large studies have reported that patients with MDD and anxiety symptoms have poorer treatment outcomes than patients who are not anxious (Wiethoff et al., 2010; Farabaugh et al., 2012; Fava et al., 2008; Ionescu et al., 2014). Although monotherapy with antidepressant treatments (ADTs) can be effective in treating MDD with anxiety symptoms, patients may be less likely to experience sustained response or remission (Ionescu et al., 2014). Subgroup analyses of short‐term, larger studies have indicated that augmentation with an antipsychotic is an effective strategy in the treatment of MDD with anxiety symptoms (Bandelow et al., 2014; Trivedi et al., 2008). Pooled analysis from two double‐blind, placebo‐controlled studies in patients with MDD and inadequate response to ADTs demonstrated that augmentation with aripiprazole improved Montgomery–Åsberg Depression Rating Scale (MADRS) total score in a subgroup with baseline Hamilton Rating Scale for Depression (HAM‐D) anxiety/somatization factor score ≥ 7 (Trivedi et al., 2008). Quetiapine extended‐release (XR) augmentation has also been reported to improve MADRS total score in subgroups of patients with MDD and inadequate response to ADTs, with anxious depression defined as baseline HAM‐D anxiety/somatization factor score ≥ 7, and alternatively as baseline Hamilton Rating Scale for Anxiety (HAM‐A) ≥ 20 (Bandelow et al., 2014).

Brexpiprazole is a serotonin–dopamine activity modulator that is a partial agonist at serotonin 5‐HT_1A_ and dopamine D_2_ receptors at similar potency, and an antagonist at 5‐HT_2A_ and noradrenaline alpha _1B/2C_ receptors (Maeda et al., 2014). In two pivotal Phase III, double‐blind, placebo‐controlled studies, brexpiprazole 2 and 3 mg day^−1^ + ADT significantly improved the MADRS total score versus placebo over 6 weeks in patients with MDD and inadequate response to ADTs (Thase et al., 2015a, 2015b). The tolerability profile observed with brexpiprazole in these short‐term studies was consistent with its receptor pharmacology. Brexpiprazole has low intrinsic activity at D_2_ receptors (Maeda et al., 2014), which may reduce the potential for activating side effects.

The objective of this Phase IIIb study (ClinicalTrials.gov NCT02013531) was to explore the effectiveness, safety, and tolerability of brexpiprazole + ADT in patients with MDD and anxiety symptoms who had an inadequate response to their current ADT.

## 2 Methods

### 2.1 Study participants

Study participants selected for this study were male or female outpatients, aged 18–65 years, who had a single or recurrent nonpsychotic episode of MDD as defined by *the Diagnostic and Statistical Manual of Mental Disorders*, Fourth edition, text revision (American Psychiatric Association, 2000), and confirmed by Mini International Neuropsychiatric Interview (Sheehan et al., 1998) and clinical examination. During the current episode, participants must have had an inadequate response, defined as <50% reduction in symptoms self‐reported via the Massachusetts General Hospital Antidepressant Treatment Response Questionnaire (MGH‐ATRQ; Chandler et al., 2010), to adequate trials of between one and three ADTs. During the 6 weeks prior to screening, participants must have received treatment at an adequate dose with one of the following single selective serotonin reuptake inhibitors (SSRIs) or serotonin noradrenaline reuptake inhibitors (SNRIs): escitalopram 10–20 mg day^−1^, fluoxetine 20–40 mg day^−1^, paroxetine controlled‐release (CR) 25–50 mg day^−1^, sertraline 50–200 mg day^−1^, duloxetine 40–60 mg day^−1^, or venlafaxine extended‐release (XR) 75–225 mg day^−1^. Eligible participants had a Hamilton Depression Rating Scale 17‐item (HAM‐D‐17; Hamilton, 1960), total score ≥ 18, and HAM‐A (Hamilton, 1959) total score ≥ 20 at both screening and baseline.

Key exclusion criteria included treatment during the current episode with an antipsychotic as adjunctive to an ADT for ≥3 weeks, electroconvulsive therapy (ECT) or transcranial magnetic stimulation; previous inadequate response to ECT or transcranial magnetic stimulation; previous vagus nerve stimulation or implantation of deep brain stimulation; new‐onset psychotherapy within 42 days prior to screening; hospitalization within 4 weeks prior to screening; other psychiatric diagnosis; hallucinations, delusions, or psychotic symptoms in current episode; serious risk of suicide; substance abuse or dependence; and significant medical condition, or abnormal laboratory tests or electrocardiogram (ECG).

If clinically appropriate, participants taking neuroleptics, monoamine oxidase inhibitors, benzodiazepines, or hypnotics were required to discontinue these medications during the screening period. Treatment with other psychotropic or investigational agents, CYP2D6 inhibitors (other than fluoxetine, paroxetine, and duloxetine), CYP3A4 inhibitors or inducers, and barbiturates was discontinued at least 24 hr before the first dose of study medication. Short‐term use of oral benzodiazepines (maximum dose: lorazepam 2 mg week^−1^ or oxazepam 30 mg week^−1^) or nonbenzodiazepine sleep aids (maximum 7 days) was allowed during the study to manage symptoms, if necessary. Anticholinergics (maximum 4 mg day^−1^ benztropine or equivalent) or propranolol (maximum 60 mg day^−1^) were permitted for management of extrapyramidal symptoms (EPS), if required. Concomitant medication was to be avoided for at least 12 hr prior to efficacy and safety assessments.

### 2.2 Study design

**Figure 1:**
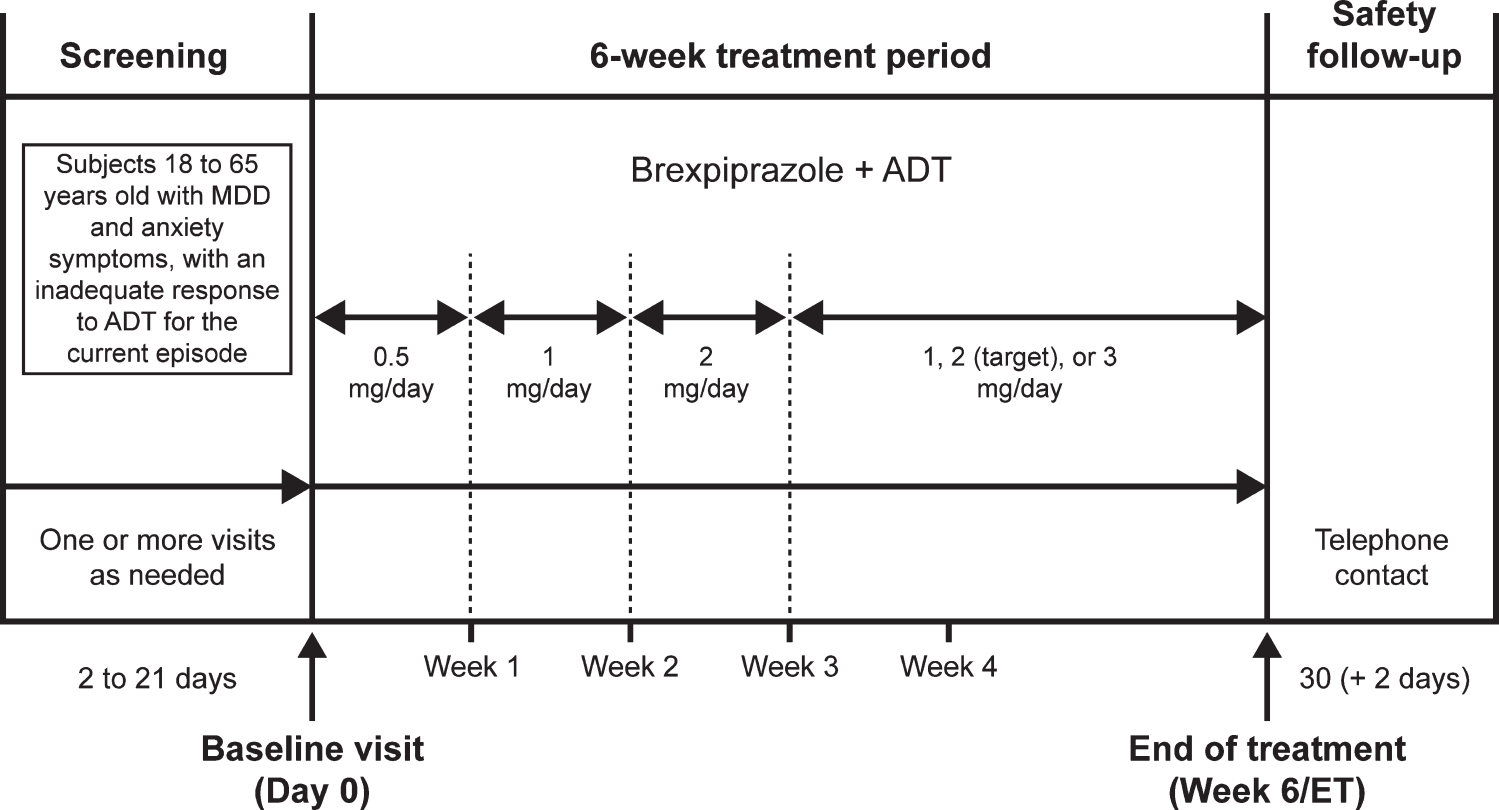
Study design. All participants received stable‐dose, open‐label SSRI or SNRI treatment throughout the screening phase. Brexpiprazole was initiated at a dose of 0.5 mg day^−1^, and was increased to 1 and 2 mg day^−1^ at Weeks 1 and 2, respectively. Thereafter, the dose could be decreased at any time to 1 mg day^−1^ or increased at study visits to 3 mg day^−1^, as required. All participants discontinued brexpiprazole at the end of the treatment phase, and were contacted by telephone to monitor safety at the end of the 30‐day follow‐up phase. ADT, antidepressant; ET, early termination; MDD, major depressive disorder; SNRI, serotonin noradrenaline reuptake inhibitor; SSRI, selective serotonin reuptake inhibitor

This was an open‐label, flexible‐dose study conducted in 12 centers in the USA between November 2013 and June 2014. The study comprised a 2–21‐day screening phase, during which participants received stable‐dose, open‐label SSRI, or SNRI treatment, a 6‐week treatment phase during which open‐label brexpiprazole (target dose 2 mg day^−1^) was added to the current ADT, and a 30‐day follow‐up phase (Fig. 1).

The study was conducted in compliance with the International Conference on Harmonization Good Clinical Practice guidelines. The protocol was approved by an institutional review board or independent ethics committee at each center. All participants provided informed consent to participate and none required surrogate consent.

### 2.3 Efficacy assessments

Efficacy was assessed using the MADRS (Montgomery & Åsberg, 1979); Clinical Global Impression‐Severity of illness scale (CGI–S) (Guy, 1976); HAM‐A; Clinical Global Impression‐Improvement scale (CGI–I) (Guy, 1976); HAM‐D‐17; Kellner Symptom Questionnaire (KSQ) (Kellner, 1987); Sheehan Disability Scale (SDS) (Sheehan, Harnett‐Sheehan, & Raj, 1996); Massachusetts General Hospital‐Cognitive and Physical Functioning Questionnaire (MGH–CPFQ) (Fava et al., 2009); and Barratt Impulsiveness Scale 11‐item (BIS‐11) (Patton, Stanford, & Barratt, 1995). MADRS, CGI‐S, HAM‐A, and CGI‐I were completed at weekly intervals from baseline to Week 6; all other assessments were conducted at baseline and Week 6 or study discontinuation. In addition, HAM‐D‐17 and HAM‐A were carried out at screening and baseline to confirm eligibility.

### 2.4 Safety and tolerability assessments

Safety and tolerability assessments were adverse events (AEs), clinical laboratory tests, body weight, vital signs, ECGs, Simpson‐Angus Scale (SAS) (Simpson & Angus, 1970), Abnormal Involuntary Movement Scale (AIMS) (Guy, 1976), Barnes Akathisia Rating Scale (BARS) (Barnes, 1989), and Columbia Suicide Severity Rating Scale (C‐SSRS) (Posner et al., 2011).

### 2.5 Statistical analysis

No formal sample size calculations were performed. The safety population comprised all participants who took at least one dose of brexpiprazole, while the efficacy population included only participants in the safety population with an efficacy assessment both at baseline and on at least one occasion postbaseline.

The primary efficacy endpoint was change from baseline at Week 6 in MADRS total score. Other efficacy endpoints included change from baseline at Week 6 in CGI–S score, HAM‐A total score, HAM‐D‐17 total score, KSQ total and symptom subscale scores, SDS mean and individual item scores, MGH‐CPFQ total score, and BIS‐11 total score, and CGI–I score at Week 6. Responder rate was defined as the proportion of participants with ≥50% reduction from baseline in MADRS total score at Week 6; or a CGI‐I score of 1 or 2 at Week 6. Remission rate was defined as the proportion of participants with MADRS total score ≤10 and a reduction from baseline of ≥50% in MADRS total score at Week 6.

Analysis of the primary efficacy endpoint was based on actual observations recorded at each visit, and no missing data were imputed (observed cases). A mixed model repeated measures (MMRM) analysis was fitted with an unstructured variance covariance structure, in which change from baseline in MADRS total score was the dependent variable. The model included fixed class effect terms for visit, baseline score, and the interaction term of score‐by‐visit. Point estimates and associated two‐sided 95% confidence intervals (CIs) were calculated. The null hypothesis of zero mean change from baseline at Week 6 was tested at a significance level of .05. As this was an exploratory trial, no methods to control type I error rate were warranted. Change from baseline at Week 6 in CGI‐S score and HAM‐A total score were analyzed in the same way as the primary efficacy endpoint. The other efficacy endpoints were summarized using last‐observation‐carried‐forward data.

Changes from baseline at last visit were reported for fasting metabolic parameters and EPS scale scores. Mean (standard deviation [*SD*]) change in body weight at Week 6 was derived from observed case data.

## 3 Results

### 3.1 Participants

We enrolled 37 participants, of whom 32 (86.5%) completed the study. Of the five participants who discontinued the study, three were lost to follow‐up, one withdrew consent, and one became pregnant and was discontinued. All 37 participants were included in the safety population; one participant was excluded from the efficacy population because they did not have an efficacy assessment both at baseline and on at least one occasion postbaseline. Participants who completed the study received brexpiprazole at a mean dose of 2.1 mg day^−1^ in addition to their current ADT.

In the safety population, mean (*SD*) age was 45.7 (15.2) years and mean (*SD*) body mass index was 29.9 (6.7) kg m^−2^ (Table 1). There were more female than male participants (female: 26/37, 70.3%), and most participants were Caucasian (26/37, 70.3%).

**Table 1:** Demographics and baseline clinical characteristics (safety population)

	Brexpiprazole, *n *= 37
Demographic characteristics	
Age, mean (*SD*), years	45.7 (15.2)
BMI, mean (*SD*), kg m^−2^	29.9 (6.7)
Gender	
Female, *n* (%)	26 (70.3)
Race, *n* (%)	
Caucasian	26 (70.3)
Black or African American	10 (27.0)
Other	1 (2.7)
Clinical characteristics	
Duration of current episode (months), mean (*SD*)	13.6 (18.0)
Recurrent depression, *n* (%)	32 (86.5)
Number of lifetime episodes, mean (*SD*)	5.5 (9.9)
MADRS total score, mean (*SD*)	30.1 (5.1)
CGI‐S score, mean (*SD*)	4.4 (0.5)
HAM‐A total score, mean (*SD*)	26.86 (5.02)
HAM‐D‐17 total score, mean (*SD*)	24.59 (3.93)
KSQ total score, mean (*SD*)	55.03 (14.52)
SDS mean score, mean (*SD*)	6.53 (1.63)
MGH‐CPFQ total score, mean (*SD*)	28.27 (4.41)

CGI‐S, Clinical Global Impression‐Severity of illness; HAM‐A, Hamilton Anxiety Rating Scale; HAM‐D‐17, Hamilton Depression Rating Scale 17‐item; KSQ, Kellner Symptom Questionnaire (range: 0–92, higher scores indicate greater number of symptoms); MADRS, Montgomery–Åsberg Depression Rating Scale; MGH‐CPFQ, Massachusetts General Hospital‐Cognitive and Physical Functioning Questionnaire (range: 7–42, higher scores indicate greater functional impairment); *SD*, standard deviation; SDS, Sheehan Disability Scale (range: 0–10, higher scores indicate greater functional impairment).

Mean (*SD*) duration of the current episode of MDD was 13.6 (18.0) months, while the median was 8.9 months. Most of the participants (32, 86.5%) had experienced recurrent episodes of depression. During the current episode, 32 participants (86.5%) reported an inadequate response to one ADT, while five participants (13.5%) had an inadequate response to at least two ADTs. None of the participants had been treated with an antipsychotic during the current episode. Baseline depression and anxiety rating scale scores confirmed that the participant population had moderate‐to‐severe depression and anxiety symptoms (Table 1).

The majority of participants were treated with an SSRI, including 15 participants (40.5%) on sertraline, 11 (29.7%) on fluoxetine, and five (13.5%) on escitalopram, while the remaining six were taking an SNRI (four [10.8%] on venlafaxine XR; two [5.4%] on duloxetine). Mean (minimum–maximum) doses during the treatment phase were as follows: sertraline 109.8 mg day^−1^ (50−200 mg day^−1^); fluoxetine 27.5 mg day^−1^ (14.1−40 mg day^−1^); escitalopram 11.5 mg day^−1^ (8.9−18.7 mg day^−1^); venlafaxine XR 159.4 mg day^−1^ (75−225 mg day^−1^); and duloxetine 60 mg day^−1^ (60 mg day^−1^). Three participants (8.1%) took lorazepam during the 6‐week treatment phase: two of these participants received 0.5 mg four times per week for 3 and 4 weeks, respectively, and one participant took a single dose of 0.5 mg.

### 3.2 Efficacy

**Figure 2:**
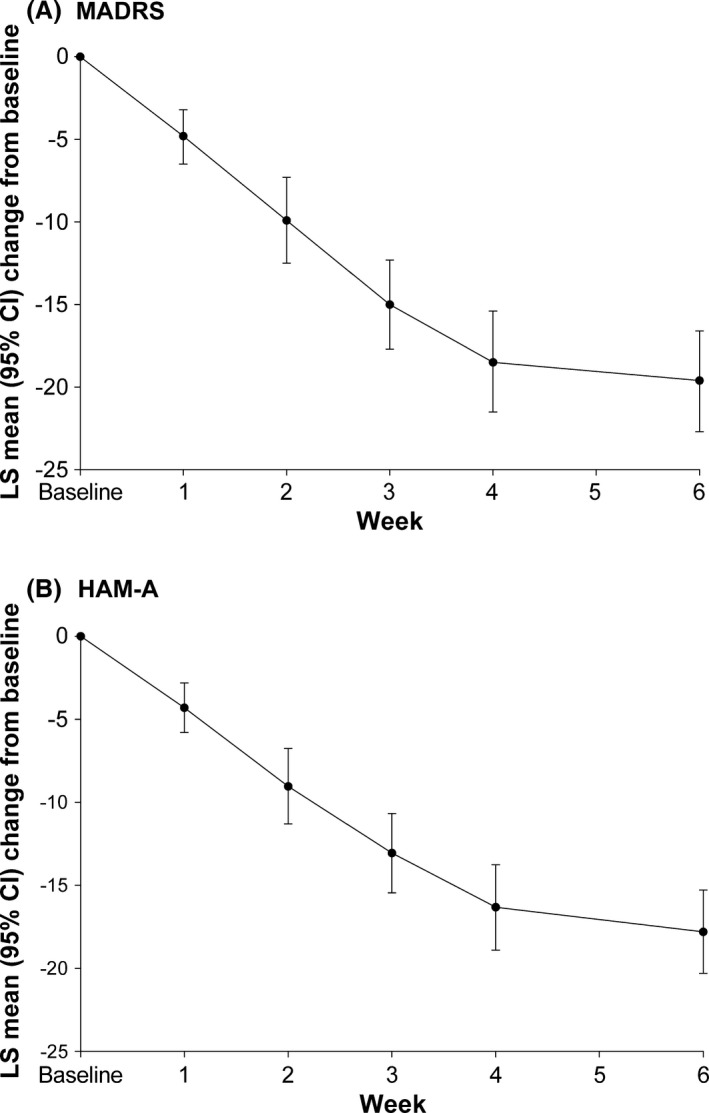
Mean (95% confidence interval) change from baseline in MADRS total score (A) and HAM‐A total score (B) during the treatment period (efficacy population). The number of participants was 36 at Week 1, 34 at Week 2, 32 at Week 3, and 33 at Weeks 4 and 6. Mean scores at baseline were 30.3 for MADRS total score and 27.0 for HAM‐A total score. CI, confidence interval; MADRS, Montgomery–Åsberg Depression Rating Scale; HAM‐A, Hamilton Anxiety Rating Scale; LS, least squares

At Week 6, improvements from baseline were seen in mean MADRS total score (least squares [LS] mean change from baseline [95% CI] −19.6 [−22.7, −16.6], *p *< .0001 vs. baseline) and HAM‐A total score (−17.80 [−20.31, −15.29], *p* < .0001). These improvements began at Week 1 and were maintained throughout the entire treatment period (Fig. 2). Significant improvements were also seen in CGI‐S, with a baseline mean (*SD*) CGI‐S score of 4.4 (0.5) reducing to 2.2 at Week 6 (−2.2 [−2.5, −1.9], *p* < .0001). Mean (*SD*) CGI‐I score at Week 6 was 1.9 (1.1).

**Figure 3:**
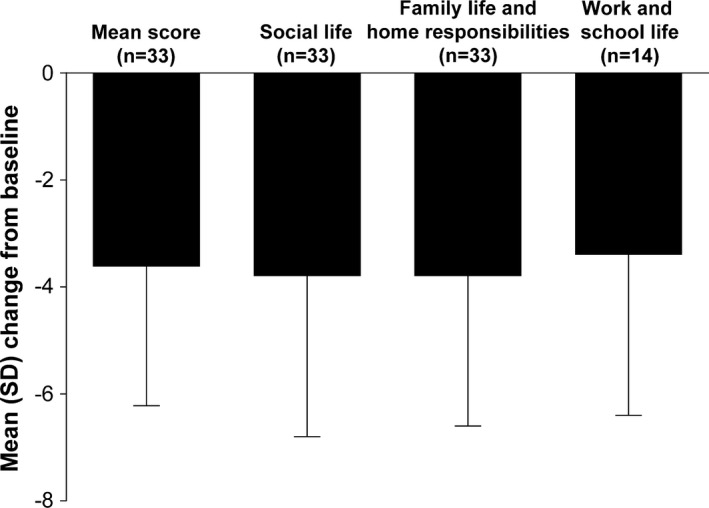
Mean (*SD*) change from baseline at Week 6 in SDS mean score and individual items (efficacy population). Mean scores at baseline were 6.5 for mean score (calculated as mean of three item scores), 6.7 for social life, 6.4 for family life and home responsibilities, and 6.3 for work and school life. Range 0–10, higher scores indicate greater impairment. *SD*, standard deviation; SDS, Sheehan Disability Scale

Mean HAM‐D‐17 total score improved from baseline at Week 6, as did MGH‐CPFQ total score and BIS‐11 total score (Table 2). Mean KSQ total score improved over the 6‐week treatment period; reductions were seen in all symptom subscale scores: anxiety, depression, somatic symptoms, and anger–hostility (Table 2). Mean (*SD*) SDS mean score improved by 3.6 (2.6) points from baseline at Week 6, while individual item scores for social life, family life, and home responsibilities, and work and school life also improved at Week 6 (Fig. 3).

**Table 2:** Mean change from baseline at Week 6 in other efficacy endpoints (efficacy population)^a^

Scale	Baseline		Change from baseline at Week 6	
	*n*	Mean (*SD*)	*n*	Mean (*SD*)
HAM‐D‐17 total score	36	24.50 (3.95)	33	−15.85 (7.37)
KSQ total score	36	55.9 (13.8)	33	−29.4 (20.9)
Symptom subscales				
Anxiety	36	10.6 (3.8)	33	−5.2 (5.1)
Depression	36	11.9 (3.4)	33	−6.6 (5.1)
Somatic symptoms	36	8.1 (4.9)	33	−4.1 (4.1)
Anger–hostility	36	8.6 (4.6)	33	−4.5 (4.5)
MGH–CPFQ total score	36	28.53 (4.18)	33	−9.85 (8.79)
BIS‐11 total score	36	71.67 (8.99)	33	−7.67 (10.14)

BIS‐11, Barratt Impulsiveness Scale 11‐item (range: 30–120, higher scores indicate greater impulsivity); HAM‐D‐17, Hamilton Depression Rating Scale 17‐item; KSQ, Kellner Symptom Questionnaire (range: total: 0–92, subscales: 0–17; higher scores indicate greater number of symptoms); MGH‐CPFQ, Massachusetts General Hospital‐Cognitive and Physical Functioning Questionnaire (range: 7–42, higher scores indicate greater functional impairment); *SD*, standard deviation.

a Last observation carried forward data.

As defined by CGI‐I, 75% (27/36) of the participants met the threshold for response at Week 6 (95% CI: 57.8%, 87.9%). As defined by MADRS, 69.4% (25/36) met the threshold for response (95% CI: 51.9%, 83.7%), and 47.2% (17/36) met the threshold for remission (95% CI: 30.4%, 64.5%) at Week 6.

### 3.3 Safety and tolerability

Treatment‐emergent adverse events (TEAEs) were reported by 75.7% (28/37) of the participants (Table 3); however, none of the TEAEs resulted in discontinuation of brexpiprazole. The most frequently reported TEAEs were increased appetite, diarrhea, dry mouth, and dizziness. Most of the TEAEs were considered by the investigators to be mild or moderate in severity, while two were considered to be severe (arthralgia; neck pain). One participant experienced a serious AE (pneumonia) after completion of brexpiprazole treatment.

**Table 3:** Adjunctive brexpiprazole treatment‐emergent adverse events (safety population, *n* = 37)

	Number of participants, *n* (%)
At least one TEAE	28 (75.7)
SAE	1 (2.7)
Discontinuation due to TEAE	0
TEAEs occurring in ≥5% of participants	
Increased appetite	5 (13.5)
Diarrhea	4 (10.8)
Dizziness	4 (10.8)
Dry mouth	4 (10.8)
Musculoskeletal stiffness	3 (8.1)
Akathisia	2 (5.4)
Arthralgia	2 (5.4)
Fatigue	2 (5.4)
Headache	2 (5.4)
Influenza	2 (5.4)
Insomnia	2 (5.4)
Irritability	2 (5.4)
Neck pain	2 (5.4)
Paresthesia	2 (5.4)
Restlessness	2 (5.4)

SAE, serious adverse event; TEAE, treatment‐emergent adverse event.

EPS‐related TEAEs were reported by 8.1% (3/37) of the participants; these were two reports of akathisia and one of extrapyramidal disorder. Both incidence of akathisia were of mild intensity; one participant had a dose reduction and the other continued on the same dose.

There were no clinically significant changes from baseline at the last visit in mean fasting metabolic parameters (Table 4). Fasting low‐density lipoprotein cholesterol changed from a normal or borderline value (<160 mg dl^−1^) at baseline to a high value (≥160 mg dl^−1^) during the treatment period for five participants, while fasting high‐density lipoprotein cholesterol changed from a normal value (≥40 mg dl^−1^) at baseline to a low value (<40 mg dl^−1^) during the treatment period for one participant. One participant recorded a change in fasting triglycerides from normal or borderline (<200 mg dl^−1^) to high (≥200 mg dl^−1^) during the treatment period. Two participants had an increase in body weight of ≥7% from baseline at Week 6; one of these participants reported weight gain as a TEAE. Mean (*SD*) increase in body weight from baseline (screening) at Week 6 was 1.6 (2.4) kg. One female participant had an elevated serum prolactin level at Week 6. In the ECG evaluations, two subjects had new‐onset QT interval >450 ms; three subjects had increases of 30–60 ms in QT interval corrected by Bazett's formula; and two participants had increases of 30–60 ms in QT interval corrected by Fridericia's formula.

**Table 4:** Mean change from baseline to last visit in fasting metabolic parameters (safety population)

Parameter	Baseline		Change from baseline at last visit	
	*n*	Mean (*SD*)	*n*	Mean (*SD*)
Cholesterol, mg dl^−1^	32	196.9 (29.7)	27	11.9 (33.0)
HDL cholesterol, mg dl^−1^	32	57.2 (13.8)	27	3.0 (10.2)
LDL cholesterol, mg dl^−1^	32	117.5 (26.9)	27	8.3 (24.7)
Triglycerides, mg dl^−1^	32	111.2 (51.3)	27	2.7 (51.8)
Glucose, mg dl^−1^	33	93.1 (10.5)	27	−0.7 (12.0)

HDL, high‐density lipoprotein; LDL, low‐density lipoprotein; *SD*, standard deviation.

There was no worsening in mean EPS scale scores. Mean (*SD*) changes from baseline at the last visit were as follows: −0.2 (0.9) for SAS total score, 0 (0) for AIMS total score, and 0 (0.2) for BARS total score. Treatment‐emergent suicidal behavior and ideation recorded on the C‐SSRS comprised one incidence of emergence of suicidal ideation and one incidence of worsening suicidal ideation.

## 4 Discussion

In this exploratory, 6‐week, open‐label study, symptoms of depression and anxiety improved in participants with MDD with concurrent anxiety who had an inadequate response to previous ADT monotherapy, following treatment with brexpiprazole at a mean dose of 2.1 mg day^−1^ adjunctive to their current ADT. Mean reductions from baseline of 20 points in MADRS total score (primary endpoint, *p* < .0001 vs. baseline) and 16 points in HAM‐D‐17 total score were observed at Week 6, together with high response (69.4%) and remission (47.2%) rates, indicating substantial improvements in symptoms of depression. These findings were supported by a reduction from baseline in CGI‐S to a mean score of 2.2 at Week 6, consistent with borderline depression and a mean CGI‐I score of 1.9 at Week 6, indicative of much improved status over baseline. Anxiety symptoms also improved substantially during the treatment period with a mean 18‐point reduction in HAM‐A total score.

In addition to improvements in clinician‐rated depression and anxiety symptoms, there was evidence of improvement over the treatment period in several other self‐rated psychiatric scale scores, including the KSQ, SDS, and MGH‐CPFQ. Improvements in KSQ symptom subscale scores indicated a reduced presence of a range of depressive, anxiety, somatic, and anger–hostility symptoms. The more than 3‐point SDS mean score change suggested considerable improvement in the study participants’ social life, family life, and home responsibilities, in addition to work and school life. The MGH‐CPFQ includes questions on motivation, wakefulness, energy, ability to focus, and mental acuity, and again, the self‐rated scores indicated that participants recognized improvements in these cognitive functions. The BIS‐11 assesses impulsive personality traits, including factors such as attention, motor control, self‐control, cognitive complexity, perseverance, and cognitive instability impulsiveness. A reduction in BIS‐11 total score was seen over the treatment period, indicating a lower level of impulsive behavior.

It is recognized that patients with MDD and anxiety symptoms may have more severe illness and may require a more intensive treatment approach (Wiethoff et al., 2010; Ionescu et al., 2014). The results from this study suggest that brexpiprazole + ADT may be an appropriate treatment option for the difficult‐to‐treat patients with MDD and anxiety. The efficacy of brexpiprazole + ADT in treating patients with MDD and an inadequate response to ADT has previously been demonstrated in two double‐blind, placebo‐controlled Phase III studies (Thase et al., 2015a, 2015b). Patients entered into the Phase III studies were not selected for a high level of anxiety as evidenced by mean baseline HAM‐A total scores of 16–18. In these studies, reductions from baseline at Week 6 in mean HAM‐A total score were greater in the 2 and 3 mg brexpiprazole + ADT groups than those in the placebo groups. In this study, mean HAM‐A total score at baseline was 27, indicating a population with a high level of anxiety. Improvements in depression and anxiety symptoms were seen as early as 1 week after initiation of brexpiprazole, and followed a similar time course.

Brexpiprazole was well tolerated in this population, consistent with previous studies (Thase et al., 2015a, 2015b). No participants discontinued due to TEAEs or reported serious AEs during treatment with brexpiprazole + ADT. The most frequently reported TEAE was increased appetite (five participants, 13.5%). Mean weight gain over the 6‐week treatment period (1.6 kg) was comparable to that reported with brexpiprazole in previous short‐term studies (Thase et al., 2015a, 2015b). Brexpiprazole did not have clinically relevant adverse effects on metabolic parameters in this short‐term study. The incidence of EPS‐related TEAEs was low, and no worsening of EPS‐related symptoms was observed using objective EPS rating scales. Few activating TEAEs were reported, with only akathisia (two participants, 5.4%) having an incidence ≥5%. This finding is clinically important since activating side effects associated with second‐generation antipsychotics (Trivedi et al., 2008) may limit their use in an anxious population. Overall, the tolerability profile of brexpiprazole observed in this study reflected its receptor pharmacology.

This study has several limitations, including the open‐label design, lack of a placebo or active comparison group, short duration of the treatment phase, heterogeneity of ADTs, and the small number of participants. Use of low‐dose benzodiazepines during the treatment period was rare; however, this may have affected the outcomes for a small number of participants. In addition, we are unable to conclude whether the improvements in anxiety symptoms were direct effects of brexpiprazole, or whether they occurred secondary to improvements in depressive symptoms.

## 5 Conclusion

In conclusion, the results of this exploratory study support the effectiveness of brexpiprazole + ADT in the treatment of depression in patients with both MDD and a high level of anxiety symptoms, a newly recognized, clinically important specifier for MDD designated “Anxious Distress” by DSM‐5 (American Psychiatric Association, 2013). The findings support the pivotal, double‐blind, placebo‐controlled studies (Thase et al., 2015a, 2015b) showing efficacy of brexpiprazole + ADT in the treatment of MDD in patients with an inadequate response to ADT monotherapy.

## Funding Information

Funding for this study was provided by Otsuka Pharmaceutical Development & Commercialization, Inc. (Princeton, USA) and H. Lundbeck A/S (Valby, Denmark). The sponsors were responsible for the study design, and the collection, analysis and interpretation of the data. The authors, some of whom are employed by the sponsors, were responsible for writing this manuscript and had responsibility for the decision to submit the article for publication.

## Conflict of Interest

The contents do not represent the views of the U.S. Department of Veterans Affairs or the United States Government. LLD has acted as a consultant to Bracket, Lundbeck, Otsuka, and Tonix; and has received research grants from Forest, Merck, and Tonix. AO and KT are employees of Otsuka Pharmaceutical Co., Ltd., Tokyo, Japan. PP and RAB are employees of Otsuka Pharmaceutical Development & Commercialization, Inc., Princeton, NJ, USA. EW is an employee of H. Lundbeck A/S, Valby, Denmark.

## References

[brb3520-bib-0001] American Psychiatric Association (2000). Diagnostic and statistical manual of mental disorders text revision (DSM‐IV‐TR), 4th ed. Arlington, VA: American Psychiatric Publishing.

[brb3520-bib-0002] American Psychiatric Association (2013). Diagnostic and statistical manual of mental disorders, 5th ed. Arlington, VA: American Psychiatric Publishing.

[brb3520-bib-0003] Bandelow, B. , Bauer, M. , Vieta, E. , El‐Khalili, N. , Gustafsson, U. , Earley, W. R. , & Eriksson, H. (2014). Extended release quetiapine fumarate as adjunct to antidepressant therapy in patients with major depressive disorder: pooled analyses of data in patients with anxious depression versus low levels of anxiety at baseline. The World Journal of Biological Psychiatry, 15, 155–166. 24506289 10.3109/15622975.2013.842654

[brb3520-bib-0004] Barnes, T. R. (1989). A rating scale for drug‐induced akathisia. British Journal of Psychiatry, 154, 672–676. 2574607 10.1192/bjp.154.5.672

[brb3520-bib-0005] Chandler, G. M. , Iosifescu, D. V. , Pollack, M. H. , Targum, S.D. , & Fava, M. (2010). RESEARCH: Validation of the Massachusetts general hospital Antidepressant Treatment History Questionnaire (ATRQ). CNS Neuroscience & Therapeutics, 16, 322–325. 19769599 10.1111/j.1755-5949.2009.00102.x PMC6493891

[brb3520-bib-0006] Farabaugh, A. , Alpert, J. , Wisniewski, S. R. , Otto, M. W. , Fava, M. , Baer, L. , … Thase, M. E. (2012). Cognitive therapy for anxious depression in STAR(*) D: What have we learned? Journal of Affective Disorders, 142, 213–218. 22877961 10.1016/j.jad.2012.04.029 PMC3483355

[brb3520-bib-0007] Fava, M. , Iosifescu, D. V. , Pedrelli, P. , & Baer, L. (2009). Reliability and validity of the Massachusetts general hospital cognitive and physical functioning questionnaire. Psychotherapy and Psychosomatics, 78, 91–97. 19218827 10.1159/000201934

[brb3520-bib-0008] Fava, M. , Rankin, M. A. , Wright, E. C. , Alpert, J. E , Nierenberg, A. A. , Pava, J. , & Rosenbaum, J. F. , (2000). Anxiety disorders in major depression. Comprehensive Psychiatry, 41, 97–102. 10741886 10.1016/s0010-440x(00)90140-8

[brb3520-bib-0009] Fava, M. , Rush, A. J. , Alpert, J. E. , Balasubramani, G. K. , Wisniewski, S. R. , Carmin, C. N , … Trivedi, M. H. (2008). Difference in treatment outcome in outpatients with anxious versus nonanxious depression: A STAR*D report. American Journal of Psychiatry, 165, 342–351. 18172020 10.1176/appi.ajp.2007.06111868

[brb3520-bib-0010] Fichter, M. M. , Quadflieg, N. , Fischer, U. C. , & Kohlboeck, G. (2010). Twenty‐five‐year course and outcome in anxiety and depression in the Upper Bavarian Longitudinal Community Study. Acta Psychiatrica Scandinavica, 122, 75–85. 19922523 10.1111/j.1600-0447.2009.01512.x

[brb3520-bib-0011] Guy, W. (1976). ECDEU Assessment Manual for Psychopharmacology. Rockville, MD: US Department of Health, Education and Welfare Publication (ADM): National Institute of Mental Health.

[brb3520-bib-0012] Hamilton, M. (1959). The assessment of anxiety states by rating. British Journal of Medical Psychology, 32, 50–55. 13638508 10.1111/j.2044-8341.1959.tb00467.x

[brb3520-bib-0013] Hamilton, M. (1960). A rating scale for depression. Journal of Neurology, Neurosurgery and Psychiatry, 23, 56–62. 10.1136/jnnp.23.1.56 PMC495331 14399272

[brb3520-bib-0014] Ionescu, D. F. , Niciu, M. J. , Richards, E. M. , & Zarate Jr, C. A. , (2014). Pharmacologic treatment of dimensional anxious depression: A review. Primary Care Companion for CNS Disorders, doi:10.4088/PCC.13r01621 10.4088/PCC.13r01621 PMC4195641 25317369

[brb3520-bib-0015] Kellner, R. (1987). A symptom questionnaire. Journal of Clinical Psychiatry, 48, 268–274. 3597327

[brb3520-bib-0016] Maeda, K. , Sugino, H. , Akazawa, H. , Amada, N. , Shimada,J. , Futamura, T. , … Kikuchi, T. (2014). Brexpiprazole I: In vitro and in vivo characterization of a novel serotonin‐dopamine activity modulator. Journal of Pharmacology and Experimental Therapeutics, 350, 589–604. 24947465 10.1124/jpet.114.213793

[brb3520-bib-0017] Montgomery, S. A. , Åsberg, M. (1979). A new depression scale designed to be sensitive to change. British Journal of Psychiatry, 134, 382–389. 444788 10.1192/bjp.134.4.382

[brb3520-bib-0018] Patton, J. H. , Stanford, M. S. , Barratt, E. S. (1995). Factor structure of the Barratt impulsiveness scale. Journal of Clinical Psychology, 51, 768–774. 8778124 10.1002/1097-4679(199511)51:6<768::aid-jclp2270510607>3.0.co;2-1

[brb3520-bib-0019] Posner, K. , Brown, G. K. , Stanley, B. , Brent, D. A. , Yershova, K. V. , Oquendo, M. A. … Mann, J. J. (2011). The Columbia‐Suicide Severity Rating Scale: Initial validity and internal consistency findings from three multisite studies with adolescents and adults. American Journal of Psychiatry, 168, 1266–1277. 22193671 10.1176/appi.ajp.2011.10111704 PMC3893686

[brb3520-bib-0020] Rhebergen, D. , Batelaan, N. M. , de Graaf, R. , Nolen, W. A. , Spijker, J. , Beekman, A. T. F. , & Penninx, B. W. J. H. (2011). The 7‐year course of depression and anxiety in the general population. Acta Psychiatrica Scandinavica, 123, 297–306. 21294714 10.1111/j.1600-0447.2011.01677.x

[brb3520-bib-0021] Sheehan, D. V. , Harnett‐Sheehan, K. , Raj, B. A. (1996). The measurement of disability. International Clinical Psychopharmacology, 11(Suppl 3), 89–95. 10.1097/00004850-199606003-00015 8923116

[brb3520-bib-0022] Sheehan, D. V. , Lecrubier, Y. , Sheehan, K. H. , Amorim, P. , Janavs, J. , Weiller, E. , … Dunbar, G. C. (1998). The Mini‐International Neuropsychiatric Interview (M.I.N.I.): The development and validation of a structured diagnostic psychiatric interview for DSM‐IV and ICD‐10. Journal of Clinical Psychiatry, 59(Suppl 20), 22–33. 9881538

[brb3520-bib-0023] Simpson, G. M. , Angus, J. W. (1970). A rating scale for extrapyramidal side effects. Acta Psychiatrica Scandinavica, 212, 11–19. 4917967 10.1111/j.1600-0447.1970.tb02066.x

[brb3520-bib-0024] Thase, M. E. , Youakim, J. M. , Skuban, A. , Hobart, M. , Augustine, C. , Zhang, P. , … Eriksson, H. (2015a). Efficacy and safety of adjunctive brexpiprazole 2 mg in major depressive disorder: A Phase 3, randomized, placebo‐controlled study in patients with inadequate response to antidepressants. Journal of Clinical Psychiatry, 76, 1224–1231. 26301701 10.4088/JCP.14m09688

[brb3520-bib-0025] Thase, M. E. , Youakim, J. M. , Skuban, A. , Hobart, M. , Zhang, P. , McQuade, R. D. , … Eriksson, H. (2015b). Adjunctive brexpiprazole 1 and 3 mg for patients with major depressive disorder following inadequate response to antidepressants: A Phase 3, randomized, double‐blind study. Journal of Clinical Psychiatry, 76, 1232–1240. 26301771 10.4088/JCP.14m09689

[brb3520-bib-0026] Trivedi, M. H. , Thase, M. E. , Fava, M. , Nelson, C. J. , Yang, H. , Qi, Y. , … Berman, R. M . (2008). Adjunctive aripiprazole in major depressive disorder: Analysis of efficacy and safety in patients with anxious and atypical features. Journal of Clinical Psychiatry, 69, 1928–1936. 19192475

[brb3520-bib-0027] Wiethoff, K. , Bauer, M. , Baghai, T. C. , Möller, H. J. , Fisher, R. , Hollinde, D. , … Aldi, M . (2010). Prevalence and treatment outcome in anxious versus nonanxious depression: Results from the German Algorithm Project. Journal of Clinical Psychiatry, 71, 1047–1054. 20673545 10.4088/JCP.09m05650blu

[brb3520-bib-0028] Zimmerman, M. , Chelminski, I. , Young, D. , Dalrymple, K. , Walsh, E. , & Rosenstein, L . (2014). A clinically useful self‐report measure of the DSM‐5 anxious distress specifier for major depressive disorder. Journal of Clinical Psychiatry, 75, 601–607. 24813209 10.4088/JCP.13m08961

[brb3520-bib-0029] Zimmerman, M. , McDermut, W. , Mattia, J. I. (2000). Frequency of anxiety disorders in psychiatric outpatients with major depressive disorder. American Journal of Psychiatry, 157, 1337–1340. 10910803 10.1176/appi.ajp.157.8.1337

